# Development of a Disease Model for Predicting Postoperative Delirium Using Combined Blood Biomarkers

**DOI:** 10.1002/acn3.70029

**Published:** 2025-03-17

**Authors:** Hengjun Wan, Huaju Tian, Cheng Wu, Yue Zhao, Daiying Zhang, Yujie Zheng, Yuan Li, Xiaoxia Duan

**Affiliations:** ^1^ Department of Anesthesiology The Affiliated Hospital, Southwest Medical University Luzhou Sichuan China; ^2^ Anesthesiology and Critical Care Medicine Key Laboratory of Luzhou Southwest Medical University Luzhou Sichuan China; ^3^ Operating Room The Affiliated Hospital, Southwest Medical University Luzhou Sichuan China; ^4^ Department of Anesthesiology Hejiang People's Hospital Luzhou Sichuan China

**Keywords:** blood biomarkers, cognitive decline, hyperlipidemia, machine learning, postoperative delirium

## Abstract

**Objective:**

Postoperative delirium, a common neurocognitive complication after surgery and anesthesia, requires early detection for potential intervention. Herein, we constructed a multidimensional postoperative delirium risk‐prediction model incorporating multiple demographic parameters and blood biomarkers to enhance prediction accuracy.

**Methods:**

We included 555 patients undergoing radical surgery for colorectal cancer. Demographic characteristics and lipid profiles were collected preoperatively, and perioperative anesthesia and surgical conditions were recorded; blood biomarkers were measured before and after surgery. The 3D‐CAM scale was used to assess postoperative delirium occurrence within 3 days after surgery. Patients were divided into the postoperative delirium (*N* = 100) and non‐postoperative delirium (*N* = 455) groups. Based on machine learning, linear and nine non‐linear models were developed and compared to select the optimal model. Shapley value‐interpretation methods and mediation analysis were used to assess feature importance and interaction.

**Results:**

The median age of the participants was 65 years (interquartile range: 56–71 years; 57.8% male). Among the 10 machine‐learning models, the random forest model performed the best (validation cohort, area under the receiver operating characteristic curve of 0.795 [0.704–0.885]). Lipid profile (total cholesterol, triglycerides, and trimethylamine‐N‐oxide) levels were identified as key postoperative delirium predictors. Mediation analysis further confirmed mediating effects among total cholesterol, trimethylamine‐N‐oxide, and postoperative delirium; a nomogram model was developed as a web‐based tool for external validation and use by other clinicians.

**Interpretation:**

Blood biomarkers are crucial in predicting postoperative delirium and aid anesthesiologists in identifying its risks in a timely manner. This model facilitates personalized perioperative management and reduces the occurrence of postoperative delirium.

**Trial Registration:**

ChiCTR2300075723

## 1 Introduction

Postoperative delirium (POD) is a common complication following anesthesia and surgery [1]. The incidence of POD varies with surgery type, ranging from 11% in hip fracture surgery [2] to 8.2%–54.4% in gastrointestinal surgery [3] and up to 46% in cardiac surgery [4]. POD occurrence increases the risk of postoperative complications, prolongs hospital stays [5], raises medical costs in the year following surgery, and exacerbates cognitive impairment [6], potentially resulting in dementia and higher postoperative mortality rates [7]. Consequently, POD has become a critical area of research, with an urgent need for further investigation into its underlying mechanisms.

Although the exact etiology of POD remains unclear, individuals with multiple “risk factors” are more likely to experience POD [8]. Recognized risk factors for POD include age, sex, body mass index (BMI), cerebrovascular disease, and educational level. [9] However, there is a lack of accurate predictive models for clinical application [10]. Thus, identifying additional risk factors and developing precise prediction models are key to improving POD risk recognition and prevention.

The occurrence of POD is closely associated with lipid metabolism disorders [11]. With improved living standards, high‐fat diets [12] and excessive meat consumption have significantly increased blood levels of trimethylamine‐N‐oxide (TMAO) [13], which is produced by the gut microbiota from dietary precursors such as choline and L‐carnitine. These precursors are converted into trimethylamine, which enters the liver via the portal vein and is metabolized by flavin‐containing monooxygenase [14]. Our recent study published in the Journal of Clinical Anesthesia demonstrated a non‐linear relationship between total cholesterol (TC) levels and POD and that TMAO in the blood is an independent risk factor for POD [15].

Recent advances in explainable artificial intelligence have enabled the interpretation of integrated machine learning (ML) models [16, 17], offering significant potential for exploring POD risk factors. Several studies have utilized ML algorithms for POD risk identification; however, most studies rely on routine clinical data and electronic health records, which often exclude sensitive biomarkers, leading to significant bias in predictive outcomes [18, 19]. Additionally, certain studies focus on sensitive variables; however, the predictive models developed generally exhibit suboptimal performance (with areas under the receiver operating characteristic curve [AUCs] ranging from 0.62 to 0.71), failing to meet the precision required for modern clinical diagnostics and treatment [20].

In this context, we construct and validate an explainable ML risk model in this study to assess the critical role of biomarkers in predicting POD and assess the potential interactions between POD risk factors. The model's findings will provide a basis for developing individualized perioperative management strategies for patients with high‐risk POD.

## 2 Methods

### 2.1 Study Participants

Data were derived from a prospective cohort study we completed previously [15]. Data have been fully anonymized, and individual identities cannot be traced. The participants were patients who underwent surgery for colorectal cancer at the Southwest Medical University Affiliated Hospital in 2023. The inclusion criteria were: (1) patients aged ≥ 18 years; (2) patients undergoing laparoscopic surgery for colorectal cancer under combined intravenous–inhalation anesthesia; (3) American Society of Anesthesiologists (ASA) physical status classification I–III; (4) informed consent obtained from the patient or their family members; and (5) a preoperative mini‐mental state examination (MMSE) score ≥ 24. The exclusion criteria were: (1) preoperative diagnosis of neurodegenerative diseases (e.g., dementia or Parkinson's disease); (2) patients with severe cardiovascular diseases or liver and kidney dysfunction; (3) patients with visual or auditory impairments or language communication disorders; and (4) patients with psychiatric disorders or those on antipsychotic medications. This study conducted a rigorous screening process. Patient data missing key variables required for the model were excluded to ensure that the final dataset used for model development and validation was complete. The study was approved by the Ethics Committee of Southwest Medical University Affiliated Hospital (KY2023180) and registered in the Chinese Clinical Trial Registry (ChiCTR2300075723).

### 2.2 Primary Endpoint Definition and Grouping

The primary endpoint was the occurrence of POD within the first 3 days after surgery. The confusion assessment method for the 3 min diagnostic interview in Chinese (3D‐CAM‐CN) was used to identify POD. The assessment was conducted daily between 15:00 and 16:00, and patients who tested positive on the 3D‐CAM were assigned to the POD group, whereas those testing negative were assigned to the non‐POD (NPOD) group.

### 2.3 Data Collection

Preoperative and intraoperative data were collected from the hospital's electronic information system, including patients' identification number, age, sex, education level, BMI, lipid levels, and medical history (e.g., coronary heart disease, hypertension, cerebrovascular events, and diabetes). Data on the duration of surgery and anesthesia, ASA classification, and intraoperative adverse events (including hypotension, hypertension, hypothermia, hypoxemia, intraoperative blood loss, and transfusion) were also collected for each patient.

### 2.4 Biomarker Collection and Testing

The expression levels of prostaglandin‐endoperoxide synthase 2 (PTGS2), TMAO, interleukin (IL)‐1β, IL‐6, and tumor necrosis factor‐α (TNF‐α) were measured preoperatively and at 1 h after surgery. Blood samples were collected from the patients' antecubital veins (3 mL) in a resting supine position using vacuum tubes and stored at 2°C–8°C. Subsequently, the samples were centrifuged at 3000 rpm for 15 min, and the plasma was frozen at −80°C for storage. Enzyme‐linked immunosorbent assay was used to measure PTGS2 (Lot No. YMS11256‐A), TMAO (Lot No. YMS10482‐A), IL‐1β (Lot No. YMS0179‐A), IL‐6 (Lot No. YMS0121‐A), and TNF‐α (Lot No. YMS0121‐A) levels using kits from Chengdu YuanNuo Tiancheng Technology Co. Ltd. All antibodies and plates used in the tests were from the same batch to avoid batch‐to‐batch variation.

### 2.5 Feature Selection for Model Construction

**FIGURE 1:**
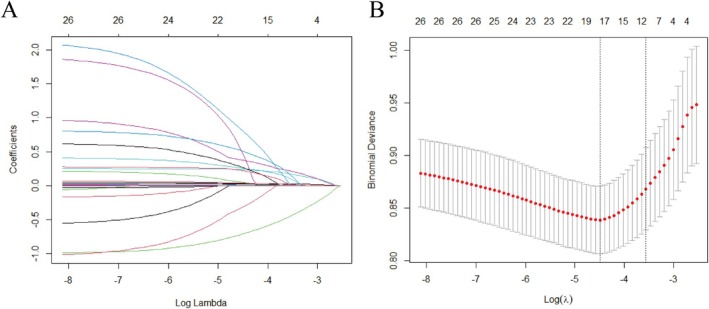
Visualization of Lasso regression. (A) The Lasso path plot shows the change in regression coefficients as the regularization parameter *λ* varies. As *λ* decreases, the model becomes less compressed, increasing its ability to select important variables. (B) The cross‐validation curve plot illustrates the average error of the model at different *λ* values. The area between the two dashed lines represents the range of ±1 standard deviation of log (*λ*).

Lasso regression was applied to the dataset for effective feature selection. The optimal regularization parameter *λ* was selected using cross‐validation, which was then used to identify the most predictive features. Ultimately, the model retained 12 features strongly correlated with the target variable, significantly improving the model's accuracy and preventing overfitting (Figure 1).

### 2.6 Model Development and Validation

In this study, we utilized 10 mL models, including linear and non‐linear models. The linear model included logistic regression, whereas the non‐linear models included support vector machine (SVM), gradient boosting machine (GBM), neural network, random forest (RF), extreme gradient boosting (XGBoost), k‐nearest neighbors (KNN), adaptive boosting (AdaBoost), light gradient boosting machine (LightGBM), and categorical boosting algorithm (CatBoost).

**FIGURE 2:**
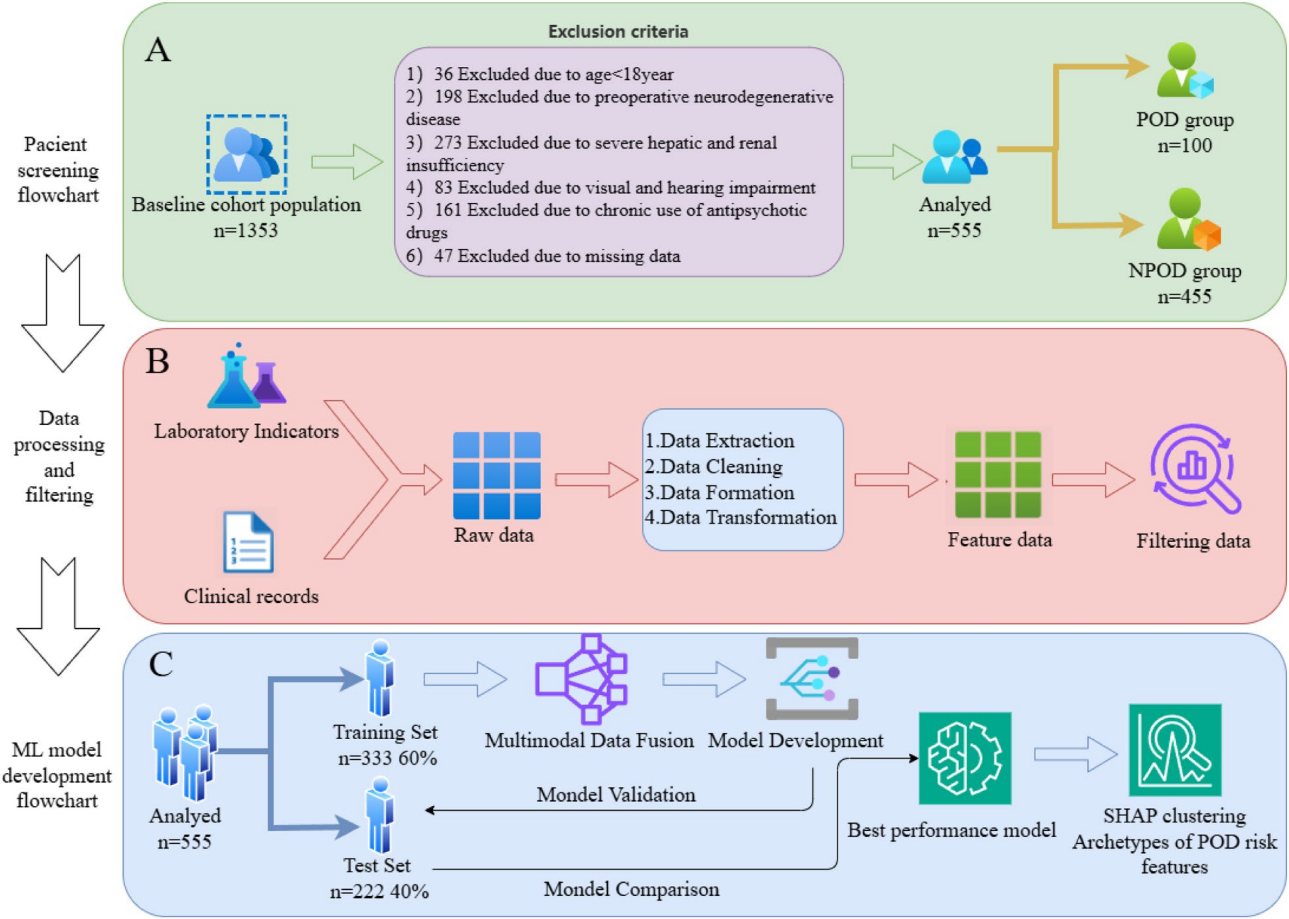
Flowchart of the study design. (A) Patient selection flowchart. (B) Data processing and filtering flowchart. (C) Development of machine learning models and visualization of important features.

The study sample was randomly divided into training (60%) and validation sets (40%). To ensure an unbiased evaluation, five‐fold cross‐validation was applied. Consequently, 10 mL models were developed in the training set through hyperparameter optimization to predict the probability of disease. Subsequently, the developed models were evaluated on the validation set, and their performance was measured using the AUC and clinical decision curve analysis (DCA). The best‐performing model was selected as the final predictive model (Figure 2).

### 2.7 Online Network Prediction Platform

A simplified ML model was developed to enhance decision‐making efficiency in assessing the risk of POD and to simplify external validation. The model used the best‐performing ML algorithms and ranks feature importance using SHapley Additive exPlanations (SHAP) values, selecting the top eight most important features. Subsequently, a nomogram model was constructed to provide an intuitive risk prediction, which was then integrated into an interactive web platform built on the Shiny framework. The platform enabled users to input relevant feature values, automatically generating the POD occurrence probability for elective surgery patients.

### 2.8 Statistical Analysis

Statistical analyses were conducted using IBM SPSS Statistics (version 28.0, Armonk, NY) and RStudio (version 4.4.1, USA). Normality tests were performed on continuous variables, with normally distributed data presented as mean ± standard deviation (SD) (*X* ± *S*) and compared between groups using an independent samples *t*‐test. Non‐normally distributed data are presented as the median and interquartile range (M [IQR]) and were compared between groups using the Mann–Whitney *U* test. Categorical data are expressed as frequency and percentage (*n* [%]) and were compared between groups using the *χ* ^2^ test or Fisher's exact test. Mediation analysis was used to explore the relationships between lipid profiles, inflammatory markers, and POD. Multiple imputation was used to handle missing data. A *p*‐value < 0.05 was considered significant.

## 3 Results

### 3.1 Patient Selection and Clinical Characteristics

#### 3.1.1 Patient Characteristics

A total of 555 patients were included in this study (Figure 2). The median age was 65 years (IQR: 56–71 years), with 57.8% of the patients being male (*n* = 321). Of all the patients, 100 (18%) were diagnosed with POD (POD group), whereas the remaining 455 patients did not experience POD (NPOD group). Compared with the NPOD group, patients in the POD group were significantly older (*p* < 0.0001). Additionally, the POD group had significantly higher levels of TC, triglycerides (TG), and low‐density lipoprotein cholesterol (LDL‐C) (*p* < 0.0001). The preoperative ASA score was also higher in the POD group (*p* < 0.0001). Further analysis showed that preoperative concentrations of TMAO (*p* < 0.05), IL‐6 levels (*p* < 0.05), and postoperative IL‐1β levels (*p* < 0.05) were significantly higher in the POD group than in the NPOD group (Table 1).

**TABLE 1:** Patient baseline and surgical characteristics.

Variables	Overall (*n* = 555)	POD (*n* = 100)	NPOD (*n* = 455)	*t*/*Z*	*p*
Preoperative situation					
Age (years)	65 (56, 71)	70 (61, 75)	64 (55, 70)	−0.025	< 0.0001
Male, *n* (%)	321 (57.8)	53 (53)	268 (58.9)	1.171	0.279
Education, *n* (%)				22.183	< 0.0001
Illiteracy or primary school	304 (54.8)	76 (76)	228 (50.1)		
Junior high school and above	251 (45.2)	24 (24)	227 (49.9)		
BMI (kg/m^2^)	22.48 ± 3.33	22.39 ± 3.34	22.50 ± 3.33	0.772	0.783
TC (mmol/L)	4.70 ± 1.07	5.12 ± 0.76	4.61 ± 1.10	−5.617	< 0.0001
TG (mmol/L)	1.28 (0.98, 1.77)	1.65 (1.35, 2.18)	1.20 (0.93, 1.67)	−6.568	< 0.0001
LDL‐C (mmol/L)	2.89 ± 0.78	3.05 ± 0.62	2.85 ± 0.80	−2.721	0.007
HDL‐C (mmol/L)	1.19 (1.01, 1.42)	1.17 (0.95, 1.41)	1.20 (1.02, 1.42)	−1.056	0.291
Coronary heart disease, *n* (%)	28 (5)	10 (10)	18 (4)	6.251	0.012
Hypertension, *n* (%)	164 (29.5)	41 (41)	123 (27)	7.682	0.006
Cerebrovascular event, *n* (%)	30 (5.4)	9 (9)	21 (4.6)	3.082	0.079
Diabetes, *n* (%)	75 (13.5)	20 (20)	55 (12.1)	4.391	0.036
MMSE	27 (26, 27)	27 (26, 27)	27 (26, 27)	−0.223	0.824
PTGS2 (ng/mL)	34.71 ± 8.50	34.17 ± 6.97	34.83 ± 8.80	0.809	0.419
TMAO (ppm)	29.03 ± 5.81	30.50 ± 6.39	28.70 ± 5.62	−2.815	0.005
IL‐1β (pg/mL)	51.56 ± 13.22	52.60 ± 14.95	51.33 ± 12.82	−0.786	0.433
IL‐6 (pg/mL)	101.62 ± 25.15	108.38 ± 22.80	100.13 ± 25.42	−2.989	0.003
TNF‐α (pg/mL)	55.08 ± 9.31	55.86 ± 9.62	54.90 ± 9.24	−0.935	0.350
ASA (≥ III)	234 (42.2%)	59 (59%)	175 (38.5%)	14.181	< 0.0001
Intraoperative conditions					
Duration of surgery > 3 h, *n* (%)	448 (40.7)	86 (86)	362 (79.6)	2.185	0.139
Duration of anesthesia > 3 h, *n* (%)	528 (95.1)	99 (99)	212 (95.9)	3.937	0.047
Intraoperative hypotension, *n* (%)	26 (4.7)	6 (6)	20 (4.4)	0.473	0.492
Intraoperative hypertension, *n* (%)	99 (17.8)	21 (21)	78 (17.1)	0.832	0.362
Intraoperative hypothermia, *n* (%)	19 (3.4)	3 (3)	16 (3.5)	0.066	0.797
Postoperative parameters					
PTGS2 (ng/mL)	53.98 ± 10.17	54.40 ± 10.04	53.88 ± 10.21	−0.457	0.648
TMAO (ppm)	40.58 (37.05, 44.38)	42.39 ± 7.17	40.60 ± 5.51	−1.934	0.053
IL‐1β (pg/mL)	79.03 (70.29, 86.85)	81.21 ± 13.41	77.70 ± 14.10	−2.336	0.019
IL‐6 (pg/mL)	150.26 ± 31.14	150.16 ± 31.89	150.29 ± 31.01	0.038	0.970
TNF‐α (pg/mL)	74.97 (68.28, 82.60)	76.67 ± 12.01	75.64 ± 12.59	−0.577	0.564

*Note:* Data are presented as mean ± standard deviation or median (interquartile range) unless otherwise specified.

Abbreviations: ASA, American Society of Anesthesiologists; BMI, body mass index; HDL‐C, high‐density lipoprotein cholesterol; IL, interleukin; LDL‐C, low‐density lipoprotein cholesterol; MMSE, mini‐mental state examination; NPOD, non‐postoperative delirium; POD, postoperative delirium; PTGS2, prostaglandin‐endoperoxide synthase 2; TC, total cholesterol; TG triglycerides; TMAO, trimethylamine N‐oxide; TNF‐α, tumor necrosis factor‐α.

### 3.2 POD Risk Prediction Model

#### 3.2.1 Comparison of Model Performance

**FIGURE 3:**
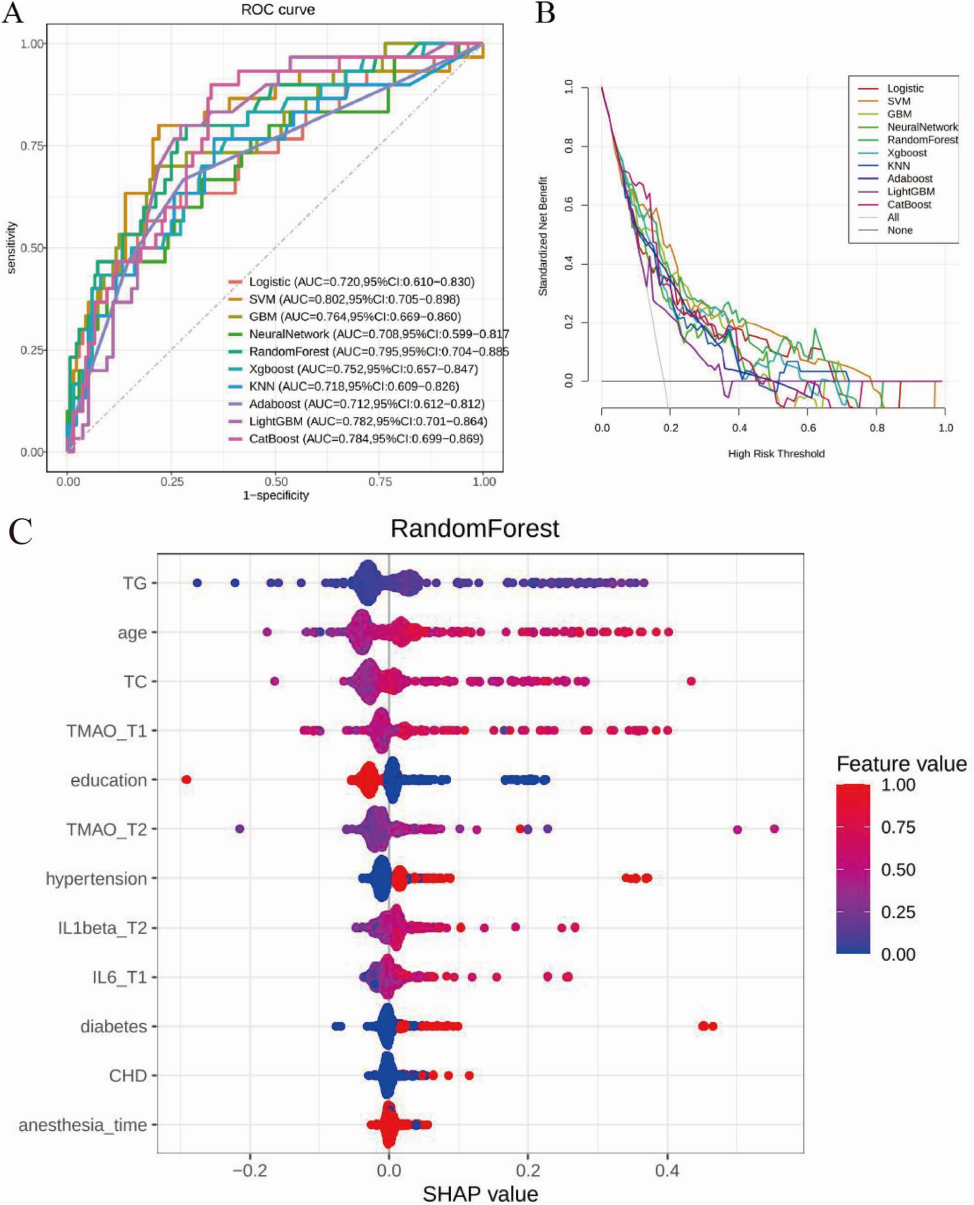
Evaluation curves of machine learning models. (A) Comparison of receiver operating characteristic curves for machine learning models. Logistic, Logistic Regression; SVM, Support Vector Machine; GBM, Gradient Boosting Machine; Neural Network, Neural Network; RandomForest, Random Forest; Xgboost, Extreme Gradient Boosting; KNN, K‐Nearest Neighbors; Adaboost, Adaptive Boosting; LightGBM, Lightweight Gradient Boosting Machine; CatBoost, Categorical Boosting Algorithm. (B) Clinical decision curve analysis. The *x*‐axis represents the probability threshold at which a clinician decides to intervene, whereas the y‐axis represents the net benefit of the decision strategy at that threshold. A higher curve indicates a more effective model. (C) SHAP Beeswarm Plot. For each predictor variable, the SHAP value levels for each patient are plotted, with the magnitude of the SHAP value represented on the *x*‐axis. The importance of each predictor variable is indicated by its total SHAP value across all patients (the predictor‐specific SHAP value). Additionally, the values of the predictor variables are represented by color: Red indicates high values, whereas blue indicates low values. For example, as age increases (from blue to red), the SHAP value increases, indicating that the likelihood of the occurrence of postoperative delirium also increases.

Based on preoperative lipid indicators (TC, TG), blood TMAO levels, and IL‐6 expression, 10 ML models were developed to predict POD. These models demonstrated significant improvements in performance, with AUC values ranging from 0.708 to 0.802 (Figure 3A). Among them, the SVM model achieved the highest AUROC value of 0.802 (0.705–0.898), followed by the RF model, with an AUROC value of 0.795 (0.704–0.885). However, DCA revealed that the RF model had a larger AUC (Figure 3B), indicating that this model offers greater potential clinical benefits. Therefore, the RF model was identified as the best predictive model for this study.

#### 3.2.2 SHAP Analysis to Identify Key Determinants of POD

The SHAP summary plot for the best‐performing RF model revealed that lipid indicators (TC, TG), perioperative TMAO levels, inflammatory markers (IL‐6, IL‐1β), age, and education level are the key variables associated with POD occurrence (Figure 3C).

#### 3.2.3 Online Prediction Platform

**FIGURE 4:**
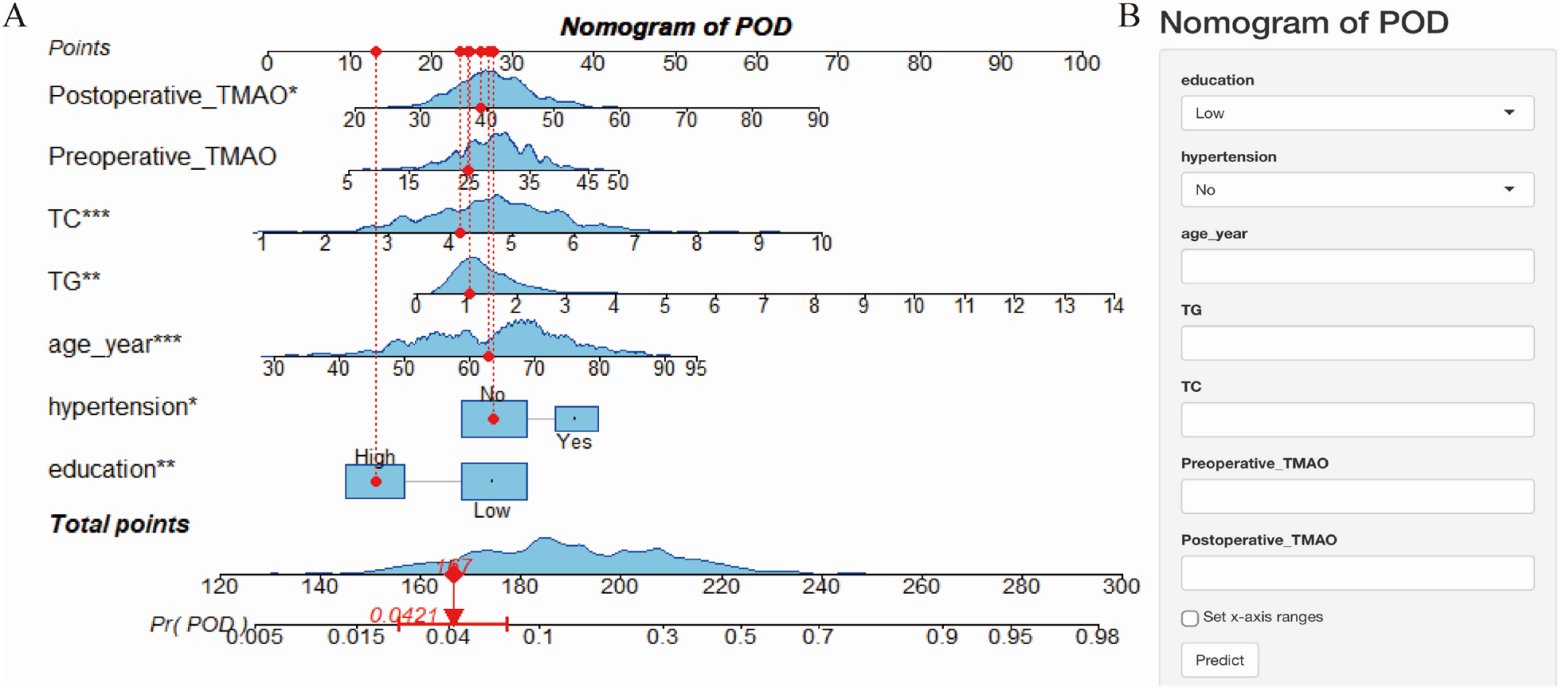
Visualization of the nomogram prediction model. (A) The nomogram model for personalized prediction of POD occurrence probability. POD, postoperative delirium; TC, total cholesterol; TG, triglycerides; TMAO, trimethylamine N‐oxide. (B) The developed online POD risk prediction tool, accessible at (https://pod‐shiny‐app.shinyapps.io/dynnomapp/).

Based on the SHAP feature importance analysis, the top seven predictive factors were selected to construct a nomogram model. An online tool was developed based on this model, designed to be user‐friendly for clinicians and to provide POD risk prediction for patients undergoing elective surgery (Figure 4).

#### 3.2.4 Mediation Analysis to Explore the Relationship Between Lipid Indicators, TMAO, and POD Outcomes

##### 3.2.4.1 Mediating Effect of TMAO Between TC and POD

**FIGURE 5:**
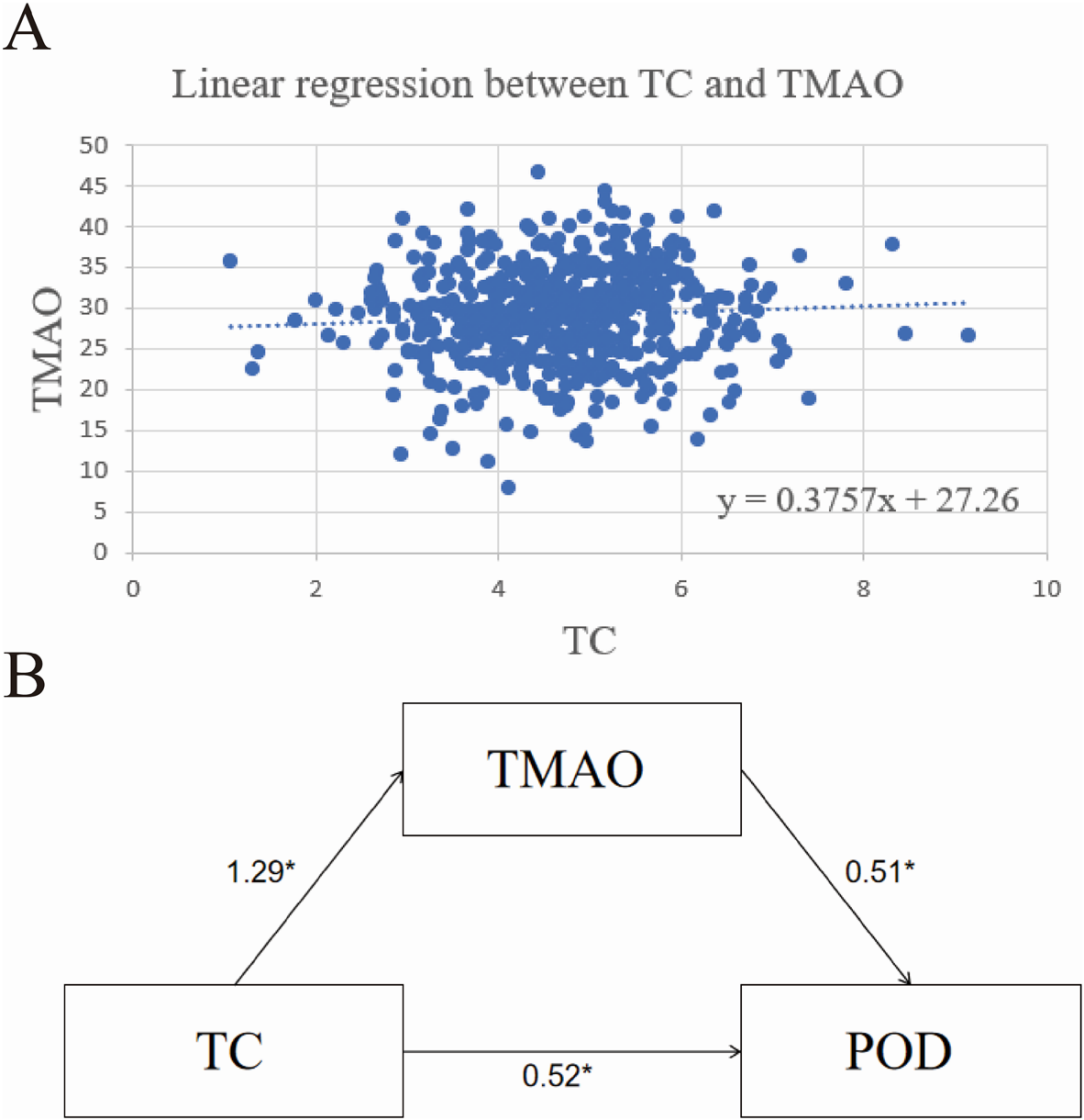
Relationship between TC, TMAO, and POD. (A) It shows the positive relationship between TC levels and preoperative TMAO concentration. Higher TC levels are associated with higher preoperative TMAO concentrations. (B) Path diagram of the relationship between TG, TMAO, and POD; 1.29 represents the effect value of TC on TMAO, 0.51 represents the effect value of TMAO on POD, and 0.52 represents the effect value of TC on POD after controlling for the mediator variable TMAO. TC, total cholesterol; TMAO, trimethylamine N‐oxide; POD, postoperative delirium. *p < 0.05.

Based on the SHAP analysis, linear regression was used to examine the relationships between biomarkers. The results showed a positive linear relationship between TC and preoperative TMAO concentration (*y* = 0.3757*x* + 27.26) (Figure 5A), which was deemed significant.

##### 3.2.4.2 Mediating Effect of Preoperative TMAO Between TC and POD

Mediation analysis revealed that preoperative TMAO significantly mediates the relationship between TC and POD incidence. Elevated TC levels may increase TMAO levels, leading to an increased risk of POD occurrence (Figure 5B).

TC directly affects POD and mediates its effect through the preoperative TMAO variable. The direct effect (0.52) and the mediating effect (0.66) account for 42.37% and 57.63% of the total effect (1.18), respectively.

## 4 Discussion

In this study, biomarkers, including lipid indices (TC, TG), perioperative TMAO levels, and inflammatory markers (IL‐6, IL‐1β), were identified as significant features for predicting POD. The study also highlights the value of using ML models to analyze perioperative variables, including biomarkers and clinical factors, especially regarding individual stress responses during surgery and anesthesia. This approach provides the potential for early identification of POD‐related risks during surgery.

Previous studies have shown promising results in using ML models to predict POD risk. In one study, researchers developed a POD prediction model based on 5 mL algorithms in older patients undergoing hip fracture surgery, identifying age as a key predictor of POD [21]. Similarly, we recognized age as a key risk factor for POD, consistent with other research findings [22, 23, 24]. Educational level, a recognized risk factor for age‐related cognitive decline, was similarly shown in this study and others to increase the likelihood of POD, with a lower educational level associated with a higher POD incidence [25, 26]. Higher educational levels may offer greater cognitive reserve, enhancing the brain's ability to process and manage damage and mitigate postoperative cognitive dysfunction [27].

In our previous study, we preliminarily identified a close relationship between TG, TC, TMAO, and POD [15]. In this study, we further confirmed the predictive value of these biomarkers for POD. However, unlike in previous studies, we used mediation analysis in our study to demonstrate that TC and preoperative TMAO levels directly affect POD, with TC also exerting an indirect effect on POD through preoperative TMAO as a mediating variable. This novel finding expands the understanding of POD risk factors and suggests potential areas for further research and therapeutic strategies.

Hyperlipidemia, with increased TG and TC levels in the blood, can disrupt the blood–brain barrier, which promotes lipid deposition in the endothelial cells of cerebral blood vessels and contributes to atherosclerotic and cerebrovascular dysfunction, thereby increasing the risk of neurodegenerative diseases [28]. Additionally, hyperlipidemia can elevate circulating TMAO levels, negatively impacting overall health [14]. Research has shown that elevated TMAO levels can induce neuronal aging, exacerbate neuroinflammation, and increase oxidative stress, thereby accelerating brain aging and cognitive dysfunction [29, 30]. Therefore, dyslipidemia and elevated TMAO levels are closely associated with POD, supporting our study's findings. However, systematic research on the relationship between hyperlipidemia, TMAO, and POD remains inadequate. Therefore, future well‐designed clinical studies, combined with animal experiments, are crucial to explore these factors and their potential biological mechanisms.

In previous research, we only used logistic regression to construct a POD prediction model [15]. By contrast, this study employed 10 ML algorithms to construct the POD prediction model, significantly enhancing the clinical applicability and generalizability of the model [31]. As ML technology is increasingly applied in the clinical field, it has become a robust tool for creating efficient risk prediction models owing to its superior data processing capabilities and predictive performance, which significantly improves prediction accuracy [32]. Researchers in numerous studies now consider ML algorithms as an effective alternative to traditional linear models (such as logistic regression or Cox regression), particularly for predicting complex clinical outcomes [33]. Additionally, we constructed a nomogram model and developed an online tool based on this model to facilitate its clinical use. This study has some limitations. First, as a single‐center study including only postoperative patients with colorectal cancer, the findings may be subject to selection bias, limiting their generalizability and external validity. Second, owing to the relatively small sample size and lack of external data validation, the statistical power and robustness of the results may be compromised. Furthermore, this study primarily focused on biomarker characteristics and did not fully consider other potentially sensitive factors affecting POD, which may introduce bias. Additionally, because patients with an MMSE score of < 24 may have mild cognitive impairment [34], we excluded this population; therefore, our model is more applicable to the preoperative population without significant cognitive impairment. Consequently, future studies will involve larger, multicenter datasets, incorporating more relevant sensitive features to optimize the accuracy and reliability of the POD prediction model.

## 5 Conclusions

Overall, the ML‐based model using biomarkers effectively predicted POD risk in surgical patients. The optimized RF model demonstrated robust classification performance and clinical applicability. Through SHAP value interpretation analysis, we identified key features that significantly contributed to POD prediction and developed a simple, interpretable nomogram model based on these features. This model has been deployed on an online platform, enabling anesthesiologists to assess individual POD risk in clinical practice and providing decision support to improve postoperative outcomes.

## Author Contributions

**Hengjun Wan**, **Huaju Tian:** data analysis and drafting of initial manuscript. **Cheng Wu**, **Yue Zhao:** patient recruitment and data collection. **Xiaoxia Duan:** study conception and design. **Xiaoxia Duan**, **Yuan Li**, **Daiying Zhang**, **Yujie Zheng:** critical revision of the manuscript for important content. All authors approval of the final manuscript.

## Conflicts of Interest

The authors declare no conflicts of interest.

## Data Availability

The datasets used and/or analyzed during the current study are available from the corresponding author on reasonable request.
